# Embracing Complexity: Peptides as Tunable Scaffolds in the Construction of Discrete Supramolecular Systems

**DOI:** 10.1002/anie.202512014

**Published:** 2025-07-25

**Authors:** Ben E. Barber, Ellen M. G. Jamieson, Leah E. M. White, Charlie T. McTernan

**Affiliations:** ^1^ Artificial Molecular Machinery Laboratory The Francis Crick Institute 1 Midland Road London NW11AT UK; ^2^ Department of Chemistry Kings College London Britannia House, 7 Trinity Street London SE1 1DB UK

**Keywords:** Peptides, Proteins, Secondary structure, Self‐assembly, Supramolecular chemistry

## Abstract

Supramolecular chemistry has advanced rapidly, with scientists using fundamental understanding to generate function from simple building blocks. However, synthetic systems are still in their infancy when compared to biology. The increasing use of peptides in supramolecular structures provides a clear roadmap to more complex function, introducing chiral, information‐rich building blocks from a readily available pool. Peptides have historically been incorporated as modular additions to discrete supramolecular architectures to interface with biological systems. More recently, supramolecular chemists have embraced the complexity of secondary and tertiary structures and peptides’ intrinsic propensity for folding to enable the formation of supramolecular architectures built from peptides, leveraging their innate properties. We explore the urgent need to embrace complex, chiral, folded building blocks in discrete supramolecular architectures and illustrate how this will provide opportunities for novel functions and applications.

## Graphical abstract

The inherent chirality, tunability, biocompatibility, and robustness of peptides provide an exciting opportunity for supramolecular chemistry. In this mini‐review, we explore uses of peptides in discrete supramolecular systems, how chemists can draw inspiration from larger biological assemblies, and discuss how embracing the complexity of peptide secondary structures in the design of these systems provides opportunities for novel structures and functions.

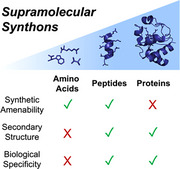

## 1 Introduction

Nature uses supramolecular chemistry to achieve almost every complex task in our bodies; from the base pairing and *π*‐stacking enabling information storage in the DNA double helix to the structure‐dictating hydrophobic core common to many proteins, supramolecular chemistry is essential to life.^[^ ^1^, ^2^, ^3^, ^4^ ^]^ The noncovalent interactions that supramolecular systems are built from are also central to many concepts in medicinal chemistry and chemical biology and the mode of action of bioactive substances. This ubiquity has inspired supramolecular chemists to try to mimic the complex and specific recognition found in natural systems. The field has evolved significantly over the years, with chemists able to precisely control the structure and functionality of discrete supramolecular assemblies. Synthetic receptors have been developed that specifically recognize biologically relevant molecules such as anions,^[^ ^5^, ^6^, ^7^, ^8^, ^9^, ^10^ ^]^ cations,^[^ ^11^, ^12^, ^13^, ^14^ ^]^ amino acids,^[^ ^15^, ^16^, ^17^, ^18^, ^19^, ^20^ ^]^ lipids,^[^ ^21^, ^22^ ^]^ peptides,^[^ ^23^, ^24^, ^25^, ^26^, ^27^, ^28^ ^]^ post‐translational modifications,^[^ ^29^, ^30^ ^]^ and sugars^[^ ^31^, ^32^, ^33^, ^34^ ^]^—in some cases matching, or even exceeding, the affinity of their biological counterparts. Artificial molecular machines have been developed to mimic the large amplitude motion and complex tasks of multiprotein complexes.^[^ ^35^, ^36^, ^37^ ^]^ Supramolecular chemistry has already had a significant impact in biomedicine, with blockbuster pharmaceuticals like Sugammadex based on supramolecular host–guest chemistry.^[^ ^38^, ^39^ ^]^ This functionalized macrocyclic sugar (a cyclodextrin) is used to reverse neuromuscular blocking agents, and its use has afforded significant improvements in patient safety since EMA approval in 2008.^[^ ^40^, ^41^ ^]^

We believe that the use of biological building blocks provides exciting avenues for the development of supramolecular chemistry in medicinal chemistry and chemical biology, which remain underdeveloped, predominantly due to two factors. First, most supramolecular chemistry occurs in organic solvents, while biological applications require stability in salty, aqueous conditions, with high concentrations of macromolecules.^[^ ^42^ ^]^ Second, interactions with biological systems require efficient interfacing with a complex, chiral, information‐rich environment, which the simple organic and planar aromatic molecules so often favored in supramolecular chemistry struggle to provide.^[^ ^43^ ^]^ Biological building blocks, such as peptides, amino acids, nucleic acids, and sugars, are often intrinsically water soluble, chiral, and able to interface with biological assemblies.^[^ ^44^, ^45^, ^46^ ^]^ The incorporation of these units in supramolecular chemistry is, therefore, highly attractive, and early uses have enabled supramolecular chemists to design systems that can interface with biology.^[^ ^33^ ^]^

As readily available biological building blocks, the 20 biogenic amino acids used to make natural proteins provide a large pool of commercially available molecules with charged, polar, and hydrophobic sidechains.^[^ ^47^ ^]^ Many of the early examples of discrete supramolecular systems used the hydrogen bonding and chirality of amino acids and short peptides in their design. The hydrogen‐bonding capabilities of peptides were of great importance in the development interlocked molecules.^[^ ^48^, ^49^, ^50^ ^]^ Goldup and coworkers have used amino acids to direct mechanical chirality.^[^ ^51^, ^52^, ^53^ ^]^ Amino acids have been used to great effect within self‐assembled metal‐organic cages to create chiral cavities.^[^ ^54^, ^55^, ^56^, ^57^ ^]^ Inspired by the self‐assembly of cyclic peptide nanotubes pioneered by Granja and Ghadiri,^[^ ^58^, ^59^, ^60^ ^]^ the hydrogen bonding capacities of amino acids and peptides have been used to form supramolecular capsules.^[^ ^61^, ^62^, ^63^, ^64^, ^65^, ^66^ ^]^ Often, however, amino acids or small peptides are used for their isolated properties such as charge, hydrogen bonding, or chirality. This is quite distinct from their critical role in biology, where the complex 3D structure of folded peptides encodes function and endows specificity.

**Figure 1:**
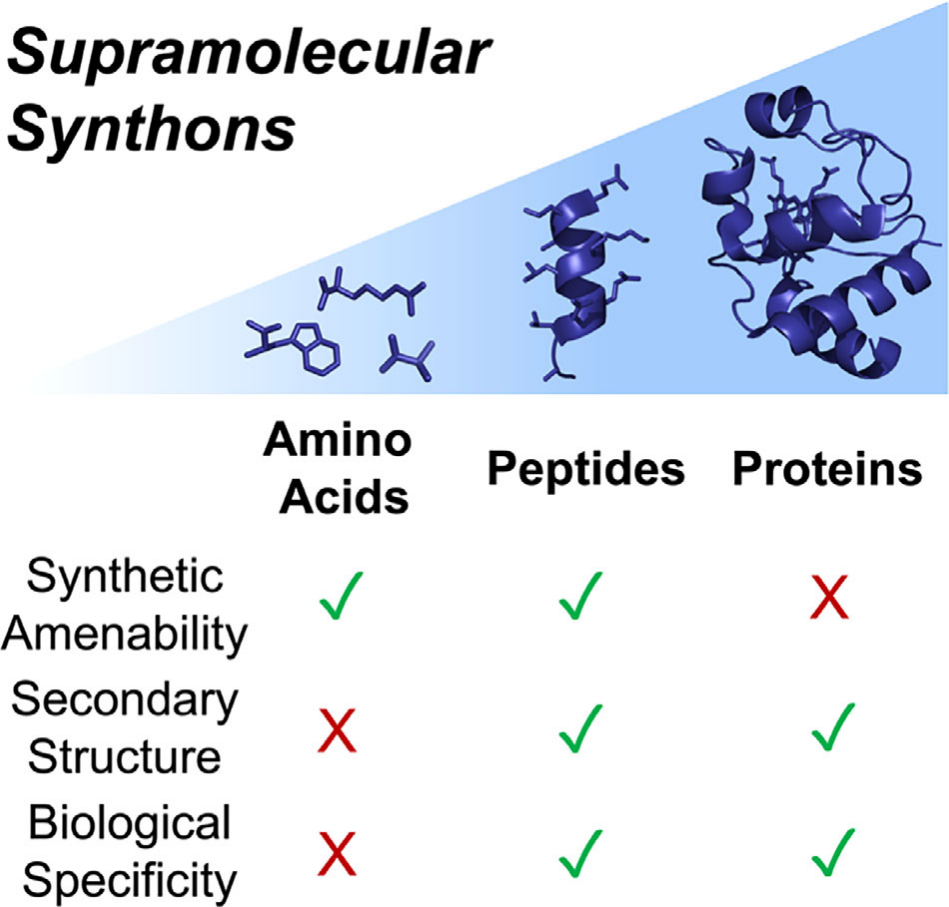
Review summary, identifying the advantages and disadvantages of applying amino acids, peptides, and proteins as building blocks in supramolecular assemblies.

Peptides, most commonly defined as oligomers of amino acids less than 50 residues in length, exhibit a wide array of secondary and tertiary structures, with folding driven by noncovalent interactions. Peptides provide access to a large and diverse area of chemical space, of which synthetic supramolecular chemists have still explored only a fraction. In this mini‐review, we will demonstrate the value of incorporating larger peptidic units to endow discrete synthetic supramolecular systems with additional biological functionality, deriving from use of a whole peptide rather than isolated amino acids or peptide fragments. We focus on recent examples whereby the intrinsic advantages of peptides are used more fully, and supramolecular systems use the folded structure and complex functions of complete peptides. This has been explored in the formation of extended (i.e., MOFs, COFs, gels, etc.) supramolecular structures and frameworks,^[^ ^67^, ^68^, ^69^, ^70^ ^]^ but is underexplored in the field of discrete supramolecular chemistry. We explore how embracing complex, chiral, and folded peptidic building blocks provides novel opportunities for structural control and functional design (Figure 1). This will enable supramolecular systems to interface with, and realize their potential in, biology and biomedicine, unlocking novel solutions to intractable challenges.

## 2 Talking to Biology—Using the Right Language

A clear rationale for the incorporation of peptides in supramolecular systems is to enable them to interface with biological systems and so provide a route to use the unique capabilities of supramolecular chemistry to address biological challenges. Endogenous peptides exhibit a wide range of functions in biology, including intra‐ and intercellular signaling, acting as defense mechanisms, and maintaining cellular homeostasis via interaction with proteins, RNA and DNA.^[^ ^71^, ^72^ ^]^ These functions require the use of functional or structural units built from multiple, rather than individual, amino acids to provide sequence‐specific information and precise interaction with biological targets. Attaching or incorporating even small peptides can therefore endow discrete supramolecular systems with enhanced biological activity.

**Figure 2:**
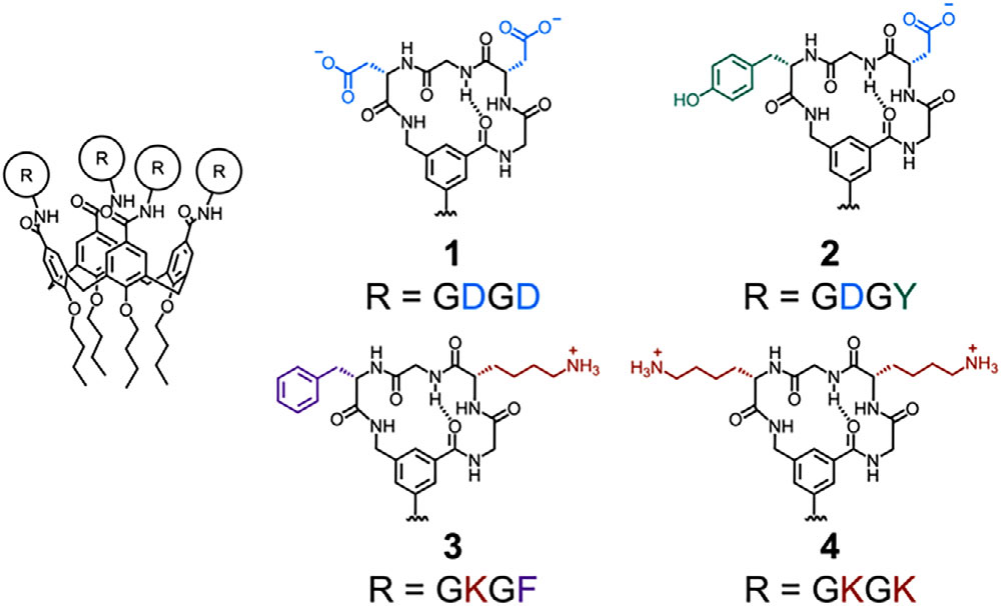
Structures of peptide‐functionalized calix[4]arenes used for targeted binding of proteins.^[^ ^74^, ^75^ ^]^

Peptide sequences often serve as specific recognition motifs in biology. Small sequences (<10 amino acids) are sufficient to generate highly selective binding interactions with much larger proteins. There are c. 100 000 unique peptide recognition motifs in biology, with their selective interactions underpinning cellular signaling, organization, communication, and regulation.^[^ ^73^ ^]^ Short peptide sequences can, therefore, serve as modules in supramolecular systems to enhance recognition of specific biomolecules of interest. Hamilton demonstrated that a calix[4]arene functionalized with four cyclic peptide loops, **1**, was able to selectively bind cytochrome C (CytC), with negatively charged aspartate (D) residues on each cyclic peptide interacting with a lysine‐rich region on the protein surface (Figure 2).^[^ ^74^ ^]^ Crucially, protein binding of the functionalized calix[4]arene was significantly enhanced compared to the free cyclic peptide, highlighting the synergistic enhancement of bringing together both calix[4]arene and cyclic peptide in a multimeric array. They later varied the peptide sequence to create a series of calix[4]arenes **2**–**4** and found **2**, containing a Gly–Asp–Gly–Tyr (GDGY) sequence in each loop, was able to strongly bind platelet‐derived growth factor and block its interaction with its receptor, interrupting signaling function and inhibiting tumor growth in mouse models.^[^ ^75^ ^]^

**Figure 3:**
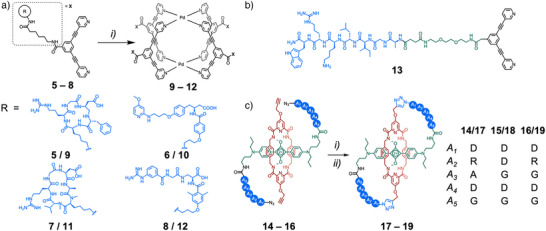
Examples of peptides used as targeting groups for discrete supramolecular systems. a) Self‐assembly of cages **9**–**12** with *exo*‐functionalized peptide/peptidomimetic ligands for integrin receptors.^[^ ^77^ ^]^ Reagents and conditions: i) Pd(MeCN)_4_(BF_4_)_2_, *d*_6_‐DMSO. b) Ligand **13** for Pd_2_L_4_ cage assembly, containing an *exo*‐blood–brain barrier translocating peptide.^[^ ^78^ ^]^ c) Squaraine‐based rotaxanes **17**–**19** as fluorescent imaging probes, containing peptide loops that alter biological localization. Reagents and conditions: i) CuBr, TBTA, Et_3_N, and CHCl_3_ and ii) TFA, TIPS, and CH_2_Cl_3_.^[^ ^79^ ^]^

The selective interactions of peptide motifs can also be used to target supramolecular systems to specific biological *loci*.^[^ ^76^ ^]^ Metal‐organic cages have shown great promise as drug‐delivery vectors for anticancer therapeutics, yet, like many oncological treatments, they are limited by off‐target toxicity. Casini, Horvatovich, Kessler, and coworkers synthesized a series of Pd_2_L_4_ cages **9**–**12** *exo*‐functionalized with peptide/peptidomimetic ligands for integrin receptors (Figure 3a).^[^ ^77^ ^]^ Multimerization led to an increased binding affinity of the ligands to their target integrin receptors once attached to the metal‐organic cage scaffold. This family of Pd_2_L_4_ cages is known to encapsulate two molecules of the anticancer therapeutic cisplatin, and cage **10** was found to enhance the cytotoxicity of cisplatin against A375 cells expressing *αvβ*3 integrin but not A549 cells (which do not express *αvβ*3 integrin). A similar *exo*‐functionalized ligand **13**, containing a known blood–brain barrier translocating peptide, encapsulated the radiolabeled ion [^99m^TcO_4_]^−^ on assembly, and penetrated the brain in in vivo mouse models (Figure 3b).^[^ ^78^ ^]^ Delivery of drugs across the blood–brain barrier remains a significant challenge, and this represents an exciting supramolecular approach to targeted cargo delivery. In both these examples, *exo*‐functionalization with targeting peptides did not perturb the host–guest chemistry of the cages, highlighting the compatibility of the two building blocks.

Smith and coworkers discovered that the encapsulation of a squaraine dye via rotaxanation greatly enhanced the chemical stability and photophysical properties of the dye.^[^ ^80^ ^]^ They, and others, have demonstrated the application of squaraine‐based rotaxanes as fluorescence imaging probes both in vitro and in vivo.^[^ ^81^, ^82^ ^]^ More recently, they reported the design of a series of self‐threaded molecular figure‐of‐eight probes **17**–**19**, comprising a squaraine‐based rotaxane motif with peptide loops linking each end of the axle to the macrocycle (Figure 3c).^[^ ^79^ ^]^ The short 4 to 5 amino acid sequences enabled the rotaxanes to display selective affinities for different locations—either the cell plasma membrane or bone. Further, the conformational restriction of the external peptidic loops enhanced their resistance to proteolytic degradation. Encapsulation of the squaraine dye in the macrocycle acts not only to enhance the performance of the fluorophore but also to minimize its impact on the targeting and pharmacokinetic properties of the external peptide loops. Both the mechanical bond, and the incorporation of peptide subcomponents are crucial elements of the design and function, working synergistically to enhance the system's properties.

**Figure 4:**
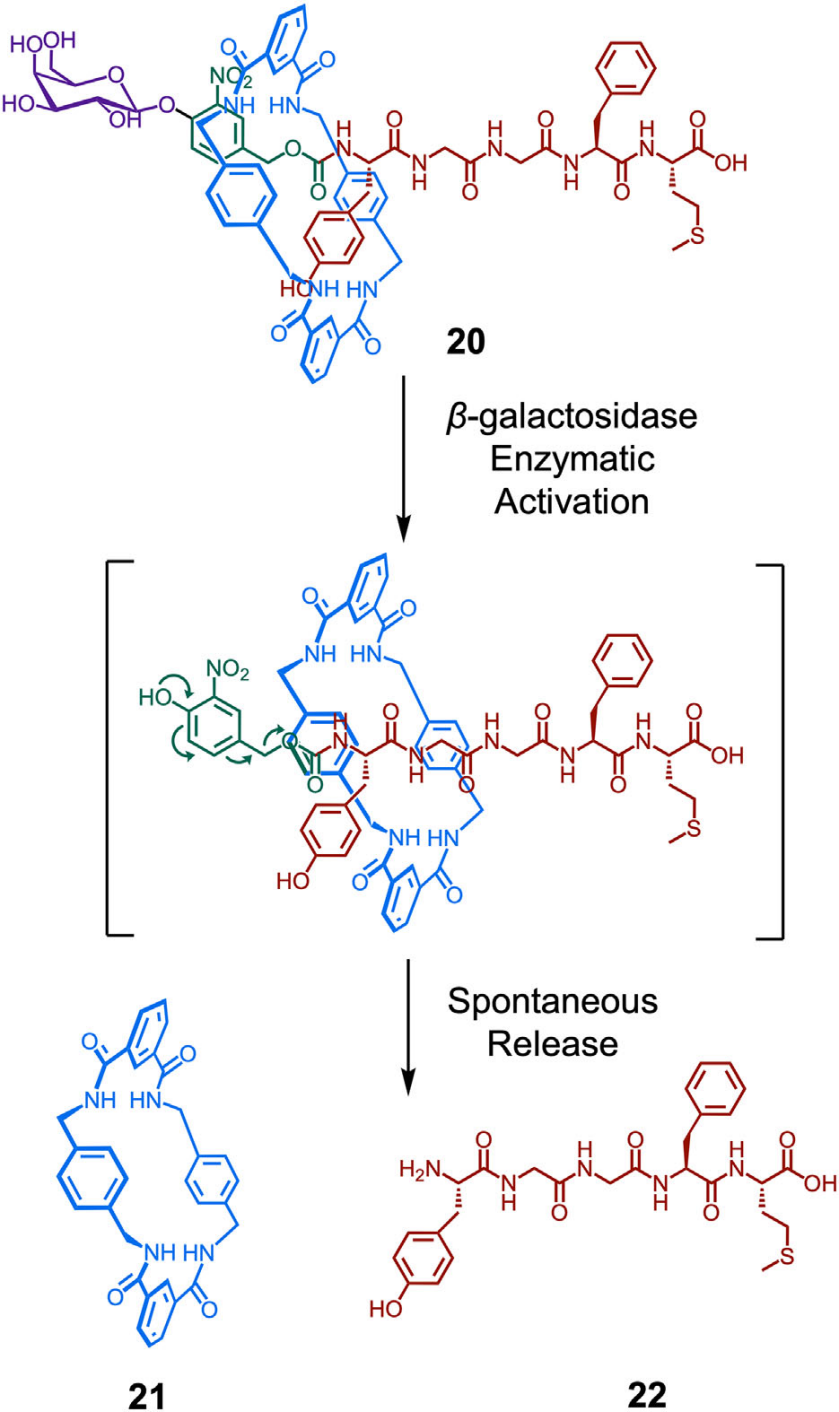
Structure of peptide‐rotaxane **20** and self‐immolative cleavage mechanism to release macrocycle **21** and free therapeutic peptide **22** upon glycosidase activation.^[^ ^90^ ^]^

Therapeutic peptides are a growing area of research interest, with over 80 peptidic drugs available on the market and more than 150 in development.^[^ ^45^ ^]^ Many naturally occurring peptides show antibacterial, antitumor and/or antiviral activity with remarkable efficacy and selectivity.^[^ ^83^, ^84^, ^85^ ^]^ Advances in rational design, peptide synthesis, and molecular biology have enabled the synthesis of a wide array of bioactive peptides.^[^ ^86^, ^87^ ^]^ Using peptide components with therapeutic activity therefore provides an avenue to address the limitations of peptides, which include their poor metabolic stability, using supramolecular therapeutics.^[^ ^88^ ^]^ Rotaxanes and pseudorotaxanes have been explored for their potential as therapeutic delivery systems. The macrocycle provides steric protection, preventing the therapeutic from engaging its target, or being degraded in the body. A specific stimulus subsequently initiates controlled disassembly of the rotaxane architecture, allowing for release of the active molecule, and so target engagement.^[^ ^89^ ^]^ Leigh, Aucagne, and Papot demonstrated this concept in the design of [2]rotaxane propeptide **20** (Figure 4).^[^ ^90^ ^]^ They synthesized a rotaxane architecture based on the peptide Met‐enkephalin **22** (H–Tyr–Gly–Gly–Phe–Met–OH), which is known to have a range of biological effects, including antitumor activity. Release of the bioactive peptide occurred via a self‐immolative mechanism, triggered by the glycosidase‐catalyzed cleavage of a carbohydrate stopper. Importantly, the macrocycle shielded the peptidic thread and significantly increased its stability to a broad range of the peptidases and enzymes found in human plasma. Given that bioactive peptides often suffer from in vivo instability, this demonstrates the unique advantages of using discrete supramolecular systems. In a second‐generation design, the authors increased the water solubility of the system via the incorporation of functional handles.^[^ ^91^ ^]^ They derivatized the parent macrocycle with two azide handles, enabling the post‐assembly attachment of hydrophilic tetra(ethylene glycol) and glucosylated tetra(ethylene glycol) chains by CuAAC. The latter was found to be 50 000 times more water soluble than the unfunctionalized structure.

**Figure 5:**
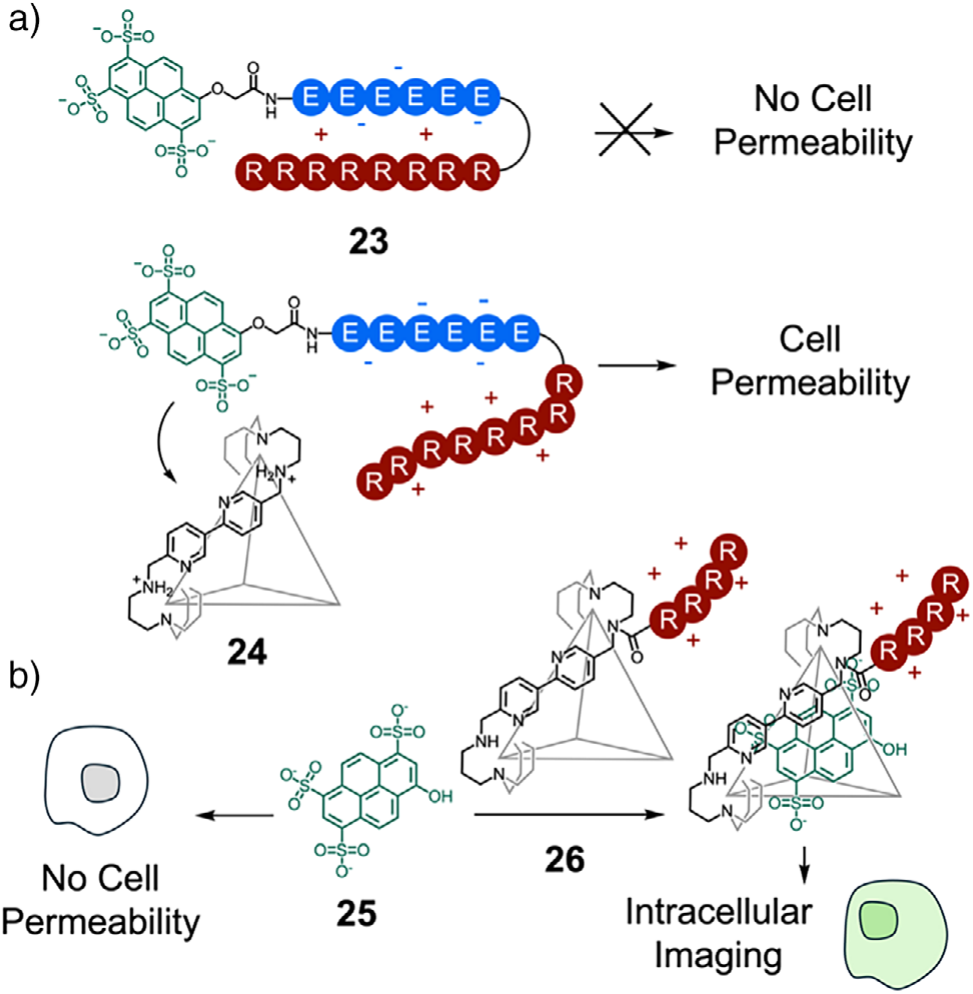
a) Schematic structure of peptide **23**, which gains cell permeability on its partial encapsulation in cage **24**.^[^ ^93^ ^]^ b) Schematic structure of cage **26**, which can make nonpenetrating anionic dyes such as pyranine **25** cell permeable.^[^ ^94^ ^]^

Transport across phospholipid bilayers, with polar exteriors and hydrophobic cores, remains a significant challenge for many therapeutic molecules. Cell‐penetrating peptides, commonly polycationic or amphipathic sequences, are frequently attached to cargo molecules to mediate cellular entry by direct translocation or endocytosis.^[^ ^92^ ^]^ The groups of Nitschke and Mascareñas reported modulation of cellular uptake of an octaarginine cell‐penetrating peptide **23** using organic cage architecture **24** (Figure 5a).^[^ ^93^ ^]^ The authors demonstrated that there was no cellular uptake of **23**, consisting of an oligoarginine domain with *N*‐terminal attachment of a polyanionic pyranine‐oligoglutamate domain. Addition of positive cage **24** to **23** created a cell permeable supramolecular complex of cage and dye. Cell viability assays confirmed that both components displayed low cytotoxicity. Importantly, neither the cage nor the pyranine‐peptide guest could cross the cell membrane individually, and they thus act synergistically upon complexation to promote efficient cellular uptake. This “supramolecular caging” strategy has been used to efficiently transport a range of nonpenetrating anionic dyes across multiple cell lines using a tetraarginine cell‐penetrating tag, attached to the exterior of the organic cage **26** (Figure 5b).^[^ ^94^ ^]^ The efficient transport of pyranine **25** into the cytosol was further used for ratiometric intracellular pH tracking.

**Figure 6:**
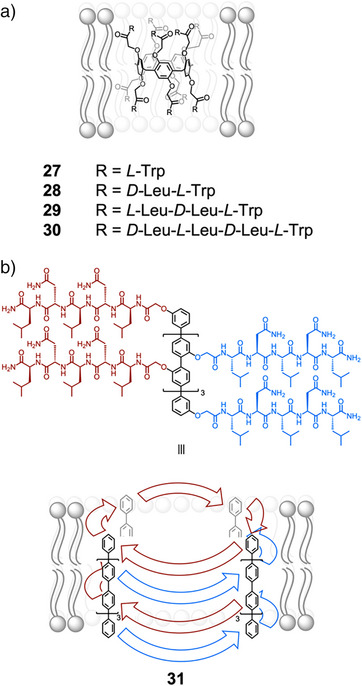
Peptides for anchoring supramolecular complexes in bilayers. a) Structures of pillar[5]arenes **27**–**30**, which form unimolecular transmembrane channels.^[^ ^96^ ^]^ b) Chemical and schematic structure of peptide‐functionalized *p*‐octaphenyl rod **31**, which self‐assembles into an artificial *β*‐barrels via interdigitation of red and blue peptide chains, and forms pores in membranes.^[^ ^98^ ^]^

While charged peptides are useful for controlling transport across membranes, hydrophobic peptide sequences provide a useful handle to anchor supramolecular complexes in phospholipid bilayers. Hou and coworkers reported the synthesis and function of a series of peptide‐appended pillar[5]‐ and pillar[6]arenes that insert in the lipid bilayer to form unimolecular transmembrane channels.^[^ ^95^ ^]^ Those containing alternating d‐ and l‐amino acids self‐assembled into confined tubular structures, induced by the intramolecular hydrogen bonding of the peptide chains of alternating chirality, and efficiently transported ions and amino acids across membranes. More recently, they incorporated Trp residues to enhance insertion selectivity **27**–**30** (Figure 6a).^[^ ^96^ ^]^ Notably, **27** exhibited high insertion selectivity for the lipid bilayer of Gram‐positive bacteria, over that of mammalian erythrocytes. As a result of this high selectivity, the channel displayed both high antimicrobial activity in Gram‐positive bacteria and low hemolytic toxicity.

Matile developed artificial *β*‐barrels formed from the self‐assembly of *p*‐octaphenyl rods functionalized with peptide sequences. The hydrogen‐bonding mediated interdigitation of peptide sequences stabilized the self‐assembly.^[^ ^97^ ^]^ The structural constraints of the *β*‐barrels led to alternating orientations of adjacent amino acid sidechains on the interior and exterior of the assembly. Therefore, by installing hydrophobic sidechains on the exterior of the assembly, such as in **31**, the *β*‐barrels could readily embed in membranes (Figure 6b).^[^ ^98^, ^99^ ^]^ This family of *β*‐barrels contain tunable cavities for binding guests and have been applied as biomimetic sensors.

These examples demonstrate the advantages of designing synergistic systems which enhance the molecular properties and function of both the supramolecular and peptidic components to achieve biologically relevant tasks. In many of these examples, the peptide serves as an attachment to, or a module within, a synthetic supramolecular system. A logical next step in the design of discrete supramolecular systems which can interface with biology is therefore to create systems made almost entirely from natural building blocks.

## 3 Building with Proteins—Discrete Supramolecular Chemistry Across Length Scales

While supramolecular chemistry typically focuses on the manipulation of systems built from small, synthetic, organic building blocks, the fundamental rules of constructing supramolecular architectures apply across length scales. The 3D tertiary folds and the associated properties of larger peptides and whole proteins can be used to synthesize discrete supramolecular systems. Protein engineering has been used to great effect to design artificial systems for applications such as drug‐delivery, vaccine development, and catalysis.^[^ ^100^, ^101^, ^102^, ^103^ ^]^

To cover this expansive and rapidly evolving field is beyond the scope of this mini‐review. Therefore, we highlight select examples in which the fundamental design principles commonly used in supramolecular chemistry are applied to build with macromolecules, to highlight similarities in approach.

Common supramolecular motifs, such as mechanical bonds, are found in natural peptides and proteins, and used as building blocks for synthetic protein systems. These structures are relatively common, with 6% of protein data bank structures forming entanglements (knots, links, and lassos).^[^ ^104^ ^]^ Link and coworkers have demonstrated that naturally occurring lasso peptides can be used as building blocks for diverse MIMs (Figure 6a).^[^ ^105^ ^]^ Two residues of the naturally occurring lasso peptide MccJ25 **32** were mutated to cysteine. On trypsin digest, this structure was then cleaved to [2]rotaxane **33** containing cysteines at each end of the thread. These building blocks associated via formation of disulfide bridges to form [3]catenane (**34**) and [4]catenane structures. Further engineering of this peptide placed cysteines at different locations, to form a dynamic combinatorial library of different MIMs, including some that had not been previously synthesized, such as [1–3]rotaxanes, [c2]daisy chains, [2–5]macrocycles, and a double‐lasso macrocycle.^[^ ^106^ ^]^

**Figure 7:**
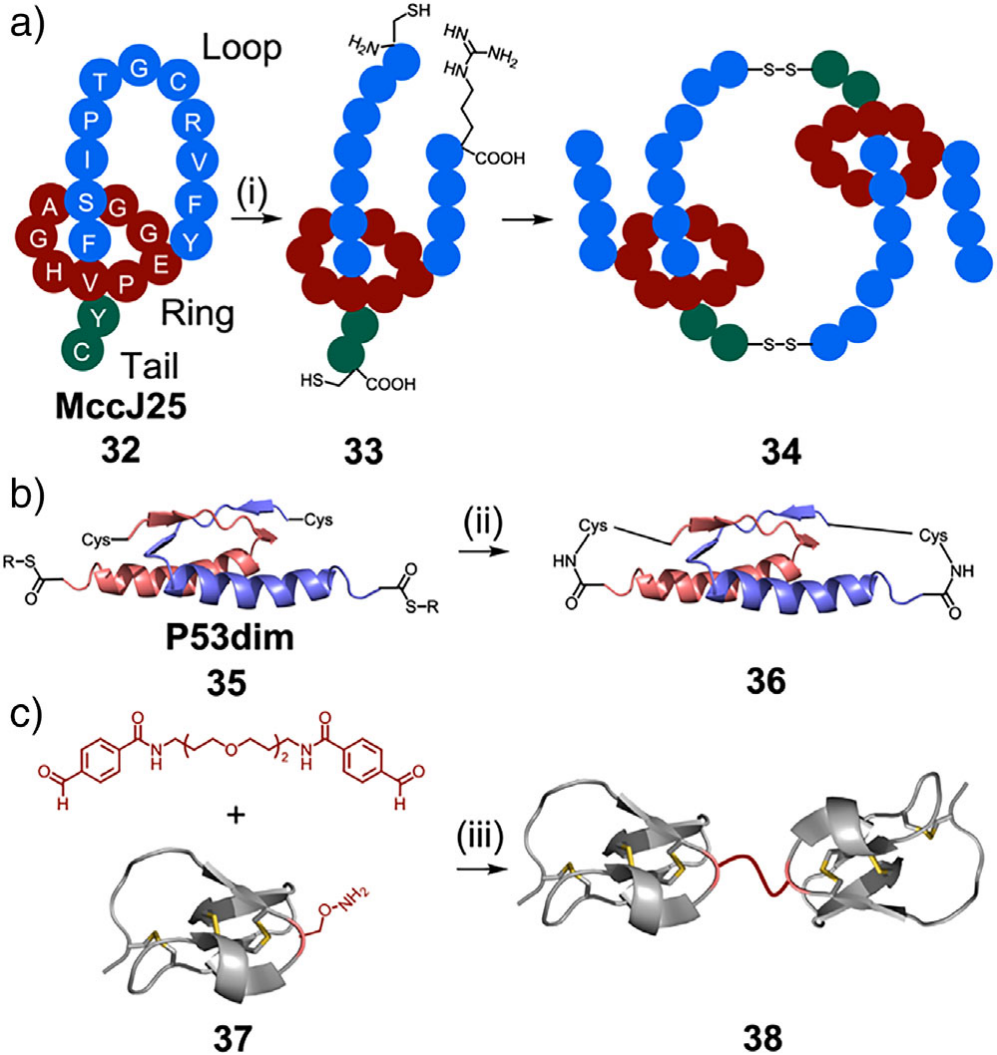
Mechanically interlocked peptides and proteins. a) Synthesis of mechanically interlocked [3]catenane using MccJ25‐derived **32**.^[^ ^105^ ^]^ Reagents and conditions: i) **32**: Trypsin, 1:70 (by mass), 50 mM NH_4_HCO_3_. b) Synthesis of protein [2]catenane cage using P53dim **35**.^[^ ^107^ ^]^ Reagents and conditions: ii) 0.1 M NaH_2_PO_4_, 1 mM EDTA, benzyl mercaptan (2 vol%), thiophenol (2 vol%). c) Synthesis of knottin dimer **38** via oxime conjugation of a chemically modified knottin EETI 2.5F monomer.^[^ ^117^ ^]^ Reagents and conditions: iii) Phosphate buffer, pH 7.4, 20 °C, 1.5 h.

Entangled peptide structures can also be engineered to produce protein catenanes. Dawson and coworkers first synthesized a protein catenane using the tetramerization domain of the tumor suppressor protein p53 (Figure 7b).^[^ ^107^ ^]^ This consisted of a dimer of dimers, p53dim **35**, in which the two proteins in each dimer are intertwined in a “bisecting U motif.” The structure can be closed by native chemical ligation to link the *N*‐ and *C*‐termini of the dimer units forming catenane **36**.

More recently, Zhang and coworkers developed an alternate method for synthesizing protein catenanes using the entwined p53dim dimer and the reaction between SpyTag and SpyCatcher sequences, which autocatalytically form isopeptide bonds, closing the catenane.^[^ ^108^ ^]^ This method could be genetically encoded, and protein catenanes were expressed in *Escherichia coli*. The sequences required for catenane formation could be fused to other proteins to generate obligate dimers and star proteins. These artificial oligomers did not dissociate at low concentrations due to the catenane linkage, and demonstrated enhanced stability to trypsin digestion. This method was further developed to produce heterocatenanes, and [3]‐ and [4]‐catenane scaffolds incorporating therapeutic affibodies (a form of antibody mimetic) such as AffiHER2.^[^ ^109^ ^]^ Catenation provides a new mechanism to synthesize “artificial antibody” scaffolds with multivalent display. The catenane incorporating AffiHER2 was compared to the wild‐type affibody, and demonstrated an increase in receptor binding affinity, longer plasma circulation times, and improved tumor accumulation. These structures serve as a bridge between fully synthetic MIMs and those found in nature, benefiting from the advantages of both paradigms.

Inhibitor cysteine knots, also known as knottins, are a structural family of highly stable miniproteins that share a common tertiary fold. Characteristically, they contain at least three interwoven disulfide bonds, which create a rigid molecular “knot” and endow these structures with exceptional chemical, thermal, and proteolytic stability.^[^ ^110^, ^111^ ^]^ Several naturally occurring knottins have found applications as therapeutics,^[^ ^112^, ^113^, ^114^ ^]^ with high loop sequence diversity underpinning a range of applications in protein engineering.^[^ ^115^ ^]^ Cochran and coworkers have shown that engineered knottins can bind with high affinity to integrin receptors, which are overexpressed on the surface of cancer cells, demonstrating their potential as molecular imaging agents.^[^ ^116^ ^]^ More recently, they developed chemically linked knottin dimer **38**, which demonstrated increased integrin receptor‐binding affinity.^[^ ^117^ ^]^ The knottin monomer **37** was chemically modified to introduce an aminooxy side‐chain, enabling dimerization by oxime ligation (Figure 7c). The dimer inhibited tumor cell migration and proliferation at nanomolar concentrations, showing greater efficacy than its monomeric counterpart, and cilengitide, an integrin‐targeting peptidomimetic, highlighting its therapeutic potential.

**Figure 8:**
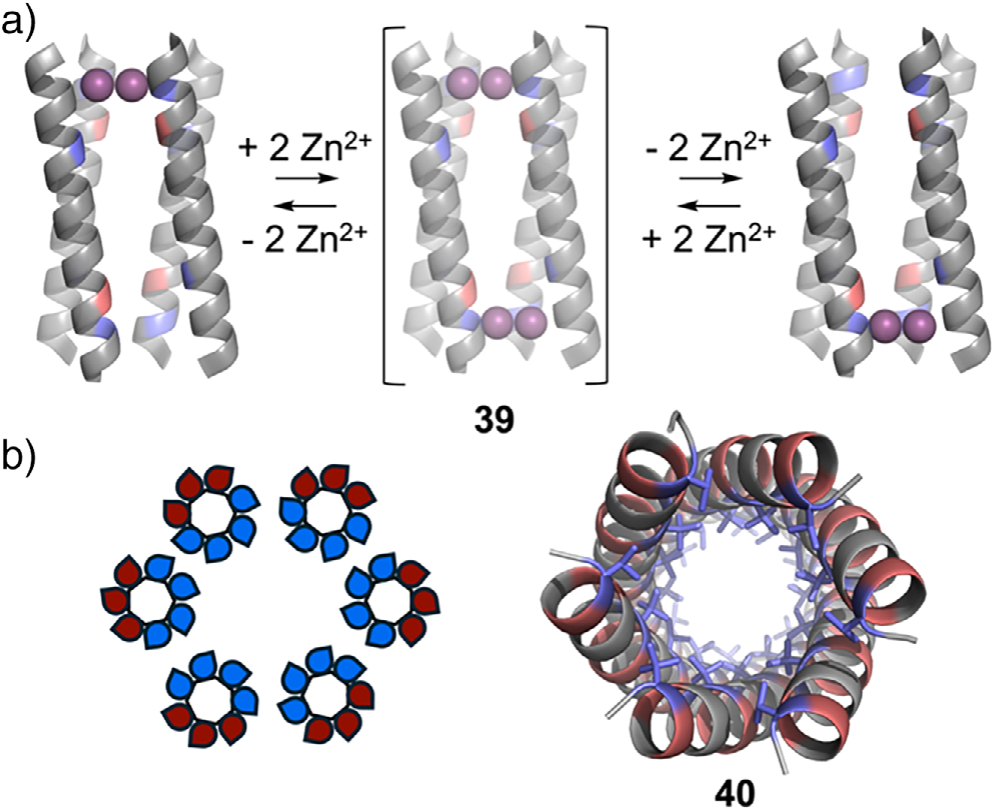
a) Schematic representation of “rocking” mechanism of **39**, a membrane spanning protein where the central symmetric state is never occupied, with Zn coordinating Glu (blue) and His (red) residues highlighted.^[^ ^120^ ^]^ b) Example helical wheel of *α*HB ion channels showing hydrophobic (red) and polar (blue) residues and crystal structure of *α*HB **40** ion channel.^[^ ^121^ ^]^

Recent years have provided significant advances in the design of membrane‐spanning peptides and proteins.^[^ ^118^ ^]^ Many of these are based on either *α*‐helical or *β*‐sheet units, forming bundled and barrel‐like structures.^[^ ^119^ ^]^ In particular, Grabe, Hong, Grigoryan, DeGrado, and coworkers reported the design of a membrane‐spanning, tetrameric coiled‐coil that selectively transports Zn^2+^ ions across membranes with concomitant reverse transport of protons.^[^ ^120^ ^]^ The authors used computational techniques to design the four‐helix bundle **39**, which contains two di‐metal binding sites that displayed negative cooperativity. Inspired by natural transporters, which are hypothesized to function by rocking between two or more states, **39** was designed to dynamically switch between two oppositely oriented symmetry‐frustrated conformational states, meaning that when Zn^2+^ was bound at one site it was released from the other (Figure 8a).

*α*‐Helical barrels (*α*HBs), a class of coiled‐coil assembly containing five or more helices, show great promise in the design of *de novo* membrane‐spanning proteins.^[^ ^119^ ^]^ Woolfson and coworkers developed a multistep approach to design *α*HB ion channels, such as **40**, that readily insert into lipid membranes.^[^ ^121^ ^]^ Having first designed peptides which could self‐assemble into water soluble *α*HBs with polar interiors and exteriors, the authors then demonstrated that modifying these to introduce outwardly facing hydrophobic residues facilitated membrane insertion (Figure 8b). The channels displayed significant activity, conducting c. 10^8^ ions per second per channel. More recently, Woolfson, Wallace, and coworkers reported the rational design of a series of pentameric, hexameric, and heptameric membrane spanning *α*HBs, focusing on the relationship between the designed coiled‐coil geometries and their ion channel conductivities.^[^ ^122^ ^]^ The authors demonstrated that computational design methods could be employed to find suitable packing solutions to stabilize *α*HBs and generate conductive channels. They noted that these peptides displayed dynamic structural changes, resulting in functional changes, highlighting both the challenges and opportunities in designing functional membrane‐spanning peptides and proteins.

Proteins can also self‐assemble to form cage‐like architectures. Well known examples include ferritin (an iron storage protein),^[^ ^123^ ^]^ encapsulins (bacterial nanocompartments),^[^ ^124^ ^]^ and viral capsids.^[^ ^125^ ^]^ Inspired by these naturally occurring polyhedral assemblies, protein engineers have designed and constructed a diverse library of structures endowed with properties beyond their natural counterparts.^[^ ^126^ ^]^ The design and application of such structures has been extensively reviewed.^[^ ^127^, ^128^ ^]^ Therefore, here we present examples that highlight the shared design principles of protein engineering and supramolecular chemistry, and show how embracing whole, functional proteins as building blocks opens avenues for exciting new applications. Successful strategies for engineering artificial protein nanocages and capsids have typically relied on using computational protein–protein interface design,^[^ ^129^ ^]^ genetic protein fusion of natively oligomeric proteins,^[^ ^130^ ^]^ and disulfide bond formation.^[^ ^131^ ^]^ However, by using coordination chemistry approaches familiar to supramolecular chemists, researchers have been able to both control and replace complex protein–protein interactions (PPIs), and so to construct artificial protein cages.^[^ ^132^, ^133^ ^]^

**Figure 9:**
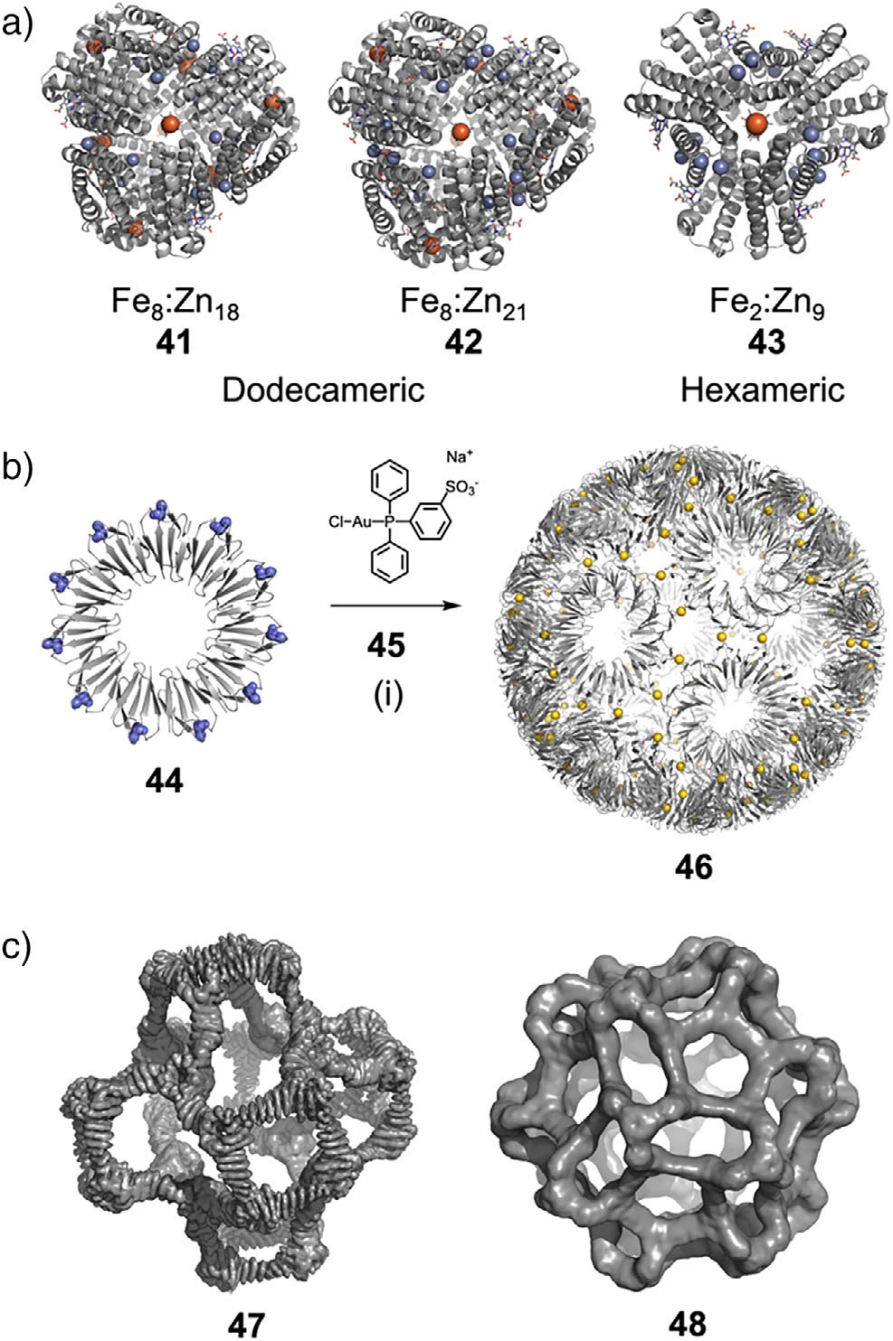
a) Crystal structures of bimetallic coordination cages **40**–**42**.^[^ ^135^ ^]^ Fe and Zn ions are represented as orange/red and blue spheres, respectively. b) Assembly of protein cage **46**, with cysteine residues of monomer **44** highlighted in blue. Au ions are represented as yellow spheres.^[^ ^136^ ^]^ Reagents and conditions: iii) **45**, 50 mM Tris‐HCl, pH 7.9, 0.15 M NaCl. c) 3D cryo‐EM maps of *T* = 4 octahedral (**47**) and icosahedral (**48**) protein cages.^[^ ^139^ ^]^

Tezcan and coworkers showed how PPIs in ferritin cage assembly could be controlled by metal coordination, forming only in the presence of chelated copper, bridging the approaches of protein interface design and supramolecular coordination chemistry.^[^ ^134^ ^]^ In another example, they demonstrated that a monomeric protein modified with biologically inspired hydroxamate groups and zinc‐binding motifs could self‐assemble into discrete dodecameric (**41** and **42**) and hexameric (**43**) cages through Fe^3+^ and Zn^2+^ coordination (Figure 9a).^[^ ^135^ ^]^ Assembly and disassembly of the cages could be mediated by chemical, thermal, and redox stimuli.

Heddle and coworkers demonstrated that the assembly of engineered protein monomer **44** with an Au(I) complex (**45**) formed an extremely stable artificial protein cage **46**, held together by Au(I) coordination between protein monomers (Figure 9b).^[^ ^136^ ^]^ They engineered trp RNA‐binding attenuation protein (TRAP), an oligomeric protein ring with an intrinsic 11‐fold rotational symmetry, such that 11 equally spaced cysteine residues were displayed along the rim (**44**). Upon addition of an Au(I)‐triphenylphosphine complex **45**, a monodisperse protein cage **46** containing 24 TRAP oligomers was synthesized. This protein cage, 22 nm in diameter, contained 120–S–Au(I)–S– “staples” between TRAP rings, which stabilized the cage to temperatures up to 95 °C and a wide pH range (3–12). The linear coordination environment of Au(I) was key to the assembly of the unusual pentagonal icositetrahedron structure of the cage—while Hg(II) also triggered cage assembly, other metals such as Cu(I), Zn(II), and Au(III) did not. These examples highlight that the fundamental principles regularly applied in supramolecular chemistry—in this case, the directional bonding approach used for metal‐organic cages—are similarly applicable in protein engineering. Therefore, we believe supramolecular chemists can likewise take inspiration from the fundamental principles of peptide and protein chemistry in their synthetic design.

Using larger proteins can also offer chemists access to unique applications, beyond the scope of small molecules. Hilvert has demonstrated that an artificial protein cage can be used for “two‐tier” supramolecular encapsulation,^[^ ^137^ ^]^ using the small porous capsid OP. This is a 13 nm diameter octahedral assembly of 24 protein monomers, containing 144 positively charged arginines lining its internal cavity. Through electrostatic interactions, this cavity could template the assembly of SDS lipids inside the cage cavity forming a hydrophobic microenvironment. These cage‐encapsulated micelles were able to encapsulate a variety of guests including the dye Nile Red. Varying the lipid composition inside the protein cage enabled tuning of the cellular delivery and serum stability of the host–guest complexes. Here, the traditional encapsulation of guests by small‐molecule supramolecular cages, often driven by hydrophobic effects, was expanded to a much larger scale using proteins.

In 2017, Ryadnov and coworkers reported the design and function of artificial virus‐like capsids that target bacterial membranes.^[^ ^138^ ^]^ The authors proposed that viral capsid architectures could serve as an ideal design platform for the development of antimicrobials. The capsids were constructed from sequences based on known host‐defense peptides (HDPs), and used the same geometric principles as naturally occurring viruses. They self‐assembled into hollow polyhedral shells of 20 nm diameter. Utilizing whole, inherently membrane‐disrupting proteins caused rapid bacterial membrane lysis. They exhibited potent antimicrobial activity towards a range of pathogenic bacteria including *Staphylococcus aureus*, *E. coli*, *Klebsiella pneumoniae*, and *Pseudomonas aeruginosa*. The development of novel antimicrobial agents is of significant scientific and global importance; however, in addition to their antimicrobial activities, these capsids could also encapsulate genetic cargo in their hollow interior, making them exciting architectures for nucleic acid delivery.

Baker and coworkers have developed novel strategies for designing more sophisticated protein nanocages by breaking nanocage symmetry.^[^ ^139^, ^140^ ^]^ Breaking symmetry to access higher triangulation number (T‐number) icosahedral structures is key to the extraordinary functionality of viruses, enabling them to efficiently encapsulate and deliver large nucleic acid cargos. Leveraging this in the construction of artificial protein cages would enable significant advances in their design and function. In a recent example, the authors developed a strategy to create protein nanocages with higher T‐numbers by breaking point group symmetry, constructing *T* = 4 cages with 48 (tetrahedral), 96 (octahedral), and 240 (icosahedral) subunits and diameters of 33, 43, and 75 nm, respectively (Figure 9c).^[^ ^139^ ^]^ The cages were constructed using pseudosymmetric heterotrimers to precisely program the six distinct protein–protein interfaces. King and coworkers described an alternate approach using pseudosymmetric oligomer building blocks to generate cage assemblies with icosahedral symmetry.^[^ ^140^ ^]^ This approach led to the formation of remarkably large nanocages, containing up to 960 subunits and with diameters up to 96 nm.

These examples serve to highlight the applicability of the fundamental design principles of supramolecular chemistry across length‐scales. While the use of large proteins is attractive for their size, inherent biological functionality, and capacity, engineering these units on scale is nontrivial, often requiring recombinant protein expression and expertise in molecular/structural biology and computational design. The size of the final assemblies may also limit certain applications. However, we believe that using smaller peptides as opposed to whole proteins—more readily accessible by solid‐phase peptide synthesis—to develop discrete supramolecular systems provides an under‐explored middle‐ground, combining the complexity and language of biology with the advantages of controlled and tunable synthetic supramolecular chemistry. Next, we explore how supramolecular chemists have begun to do this, using secondary and tertiary peptide structures to enable the formation of supramolecular architectures.

## 4 The Middle‐Ground—Embracing Peptide Secondary Structure in Supramolecular Chemistry

The use of the 3D structure and folding of synthetic peptides in the construction of discrete supramolecular systems represents the state‐of‐the‐art in the field. It provides access to complex and collaborative function, while retaining the modularity of organic synthesis. Unlike organic molecules, which can typically adopt multiple conformations of similar energy in solution, restricted rotation about peptide bonds results in a limited number of preferred, low‐energy, conformations. This can be leveraged in the design of supramolecular systems, as it leads to defined, and often predictable, secondary structure—where fulfilment of internal hydrogen bonding or electronic interactions dictates the 3D conformation of the peptide. The most famous examples are the *α*‐helix and β‐sheet. However, depending on sequence, or incorporation of nonproteinogenic and unnatural amino acids, alternative secondary structures such as 3_10_ or polyproline II (PPII) helices are readily accessible.^[^ ^141^, ^142^, ^143^ ^]^ Automated solid‐phase peptide synthesis (SPPS) allows rapid, and operationally trivial, synthesis of peptides of almost any sequence. As such, peptides provide access to a large and diverse area of chemical space, of which synthetic supramolecular chemists have explored only a fraction. Foldameric and peptoid structures can be synthesized by similar methods and share many advantages, further expanding the accessible chemical space. The design and application of these systems has been detailed elsewhere.^[^ ^144^, ^145^, ^146^ ^]^ Peptide secondary structures can be considered scaffolds in which specific functional groups are held in defined orientations from the main helical chain, providing a blueprint for the design of functional systems.

**Figure 10:**
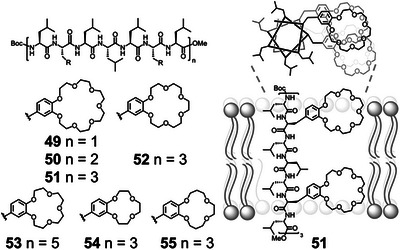
Structures (left) of artificial ion channels **49**–**55** and schematic showing insertion of **51** into a membrane with crown ether sidechains held in alignment by the *α*‐helical scaffold, which enables ion transport.^[^ ^148^ ^]^

The controlled conformations of peptide secondary structures have been used in the construction of artificial ion channels. Voyer demonstrated the use of *α*‐helical peptides **49**–**55** as scaffolds to align crown ethers (Figure 10).^[^ ^147^, ^148^ ^]^ Crown ether amino acids were prepared by derivatization of l‐3,4‐dihydroxyphenylalanine (l‐DOPA), and inserted in an *α*‐helical scaffold to present the crown ethers to the same helical face. Hydrophobic leucine residues enabled ready insertion into membranes, and the modularity of peptide synthesis allowed full exploration of how peptide length, sequence, and crown ether size affected cation transport across membranes.^[^ ^149^ ^]^

The use of peptides in metallosupramolecular chemistry offers a powerful example of how defined geometries in organic molecules can be used to build discrete supramolecular systems.

To favor discrete metal‐organic assemblies over extended polymeric systems and intractable masses, interacting functional groups and metal binding residues must be held in specific orientations. The pioneering work of Fujita and Sawada elegantly demonstrated this principle by generating metal‐peptidic interlocked molecules. Their work was inspired by the viral HK97 capsid, a remarkably thin hollow capsule with a diameter of 660 Å, formed from 420 individual peptide subunits that, upon protein self‐assembly, autocatalytically link to form a shell of interlocked rings described as “protein chainmail”.^[^ ^150^ ^]^ The metal‐peptidic structures of Fujita and Sawada were formed by a similar “Folding‐and‐Assembly” process, in which the folding of flexible peptides into organized secondary structures promoted the assembly of higher‐order species, while cooperatively the formation of higher‐order species created inter‐peptide interactions that stabilized weak intramolecular forces defining secondary structures.^[^ ^151^ ^]^

**Figure 11:**
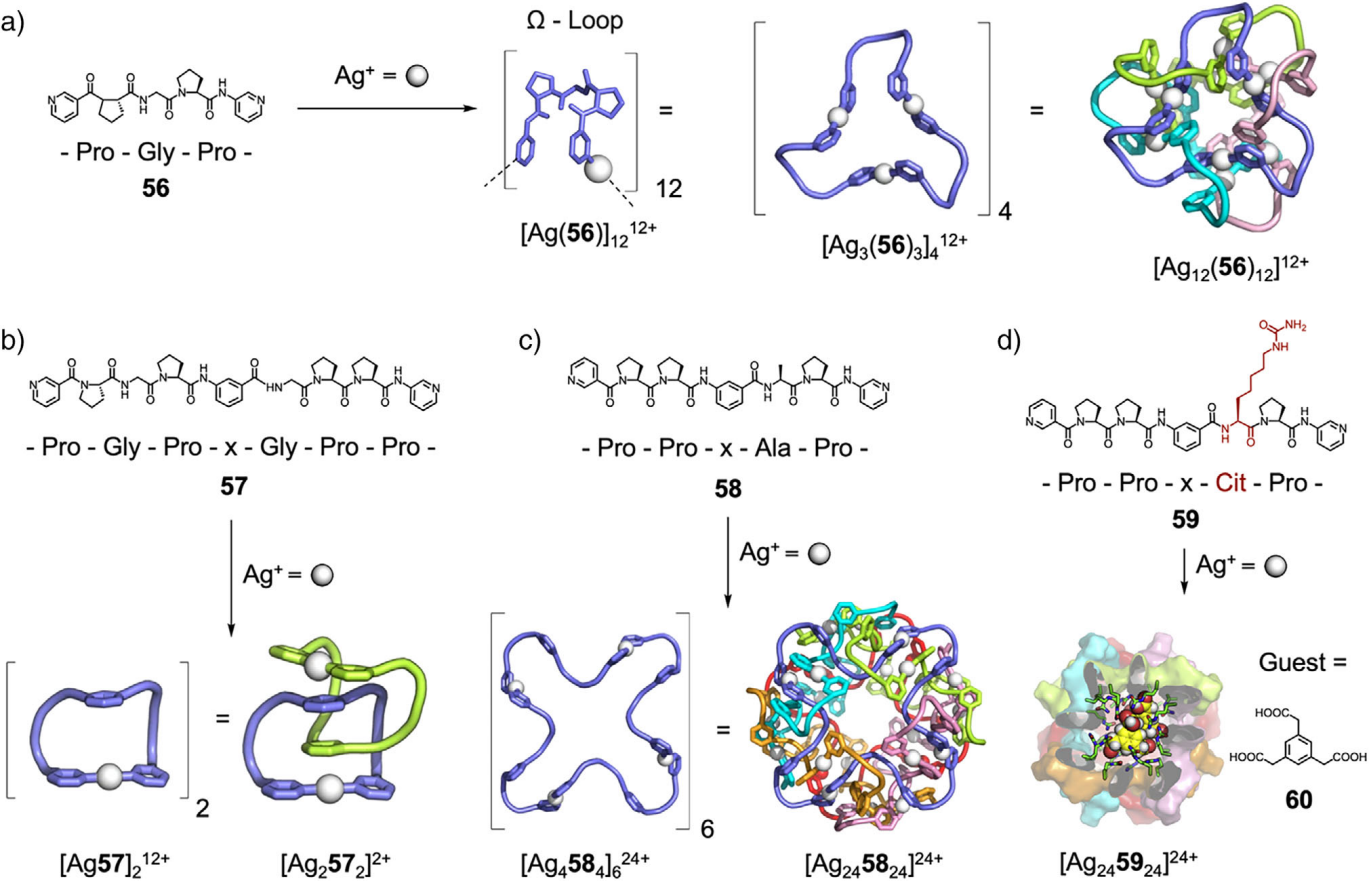
Metallosupramolecular structures using "Folding‐and‐Assembly" approach. a) Self‐assembly of **56** into a 12‐crossing [4]catenane.^[^ ^152^ ^]^ Reagents and conditions: AgBF_4_, MeOH. Adapted from Ref.[151] Copyright © 2020, Fujita et al. b) Self‐assembly of **57** into a [2]catenane.^[^ ^155^ ^]^ c) Self‐assembly of **58** into a [6]catenane capsule.^[^ ^158^ ^]^ Reagents and conditions: AgNTf_2_, CD_3_NO_2_. Adapted from Ref.[151] Copyright © 2020 Fujita et al. d) Self‐assembly of **59** into a [6]catenane capsule, suitable for binding guest **60**.^[^ ^158^ ^]^ Reagents and conditions: AgNTf_2_, CD_3_NO_2_. Adapted from Ref.[151] Copyright © 2020 Fujita et al.

The pyridyl‐appended peptide **56** contains the amino acid sequence Pro–Gly–Pro, which adopts an Ω‐loop conformation due to the flexibility of Gly and rigidity of two flanking Pro residues (Figure 11a).^[^ ^152^ ^]^ On addition of Ag(I), peptide **56** self‐assembled to form Ag_3_ **56**_3_ macrocycles which spontaneously threaded to form an Ag_12_ **56**_12_ 12‐crossing [4]catenane driven by *π* stacking between pyridines. The Ω‐loop helped preorganize the peptide's fold to promote formation of the discrete [4]catenane, as opposed to previously observed extended metal‐peptidic frameworks.^[^ ^153^, ^154^ ^]^ When the longer, analogous, peptide Pro–Gly–Pro–X–Gly–Pro–Pro (**57**) was used, complexation with Ag(I) formed a [2]catenane structure (Figure 11b).^[^ ^155^ ^]^ The “Folding‐and‐Assembly” approach has proven a powerful way to generate interwoven structures, with a M_60_L_60_ interwoven capsule approaching the size and complexity of large proteins recently reported.^[^ ^156^ ^]^

Utilizing peptidic cores enables subtle changes in a system's properties to be programmed by changing peptide sequence without significant disruption to structure, or a requirement to develop new synthetic routes. Peptide **56** was amenable to changes in peptide sequence such that similar pyridyl‐appended peptides, with sequences Ile–Pro–Pro, Val–Pro–Pro, Thr–Pro–Pro, and Ala–Pro–Pro, formed PPII conformations in assembled structures, with a “loop” geometry analogous to the previously reported Ω‐loop.^[^ ^157^ ^]^

**Figure 12:**
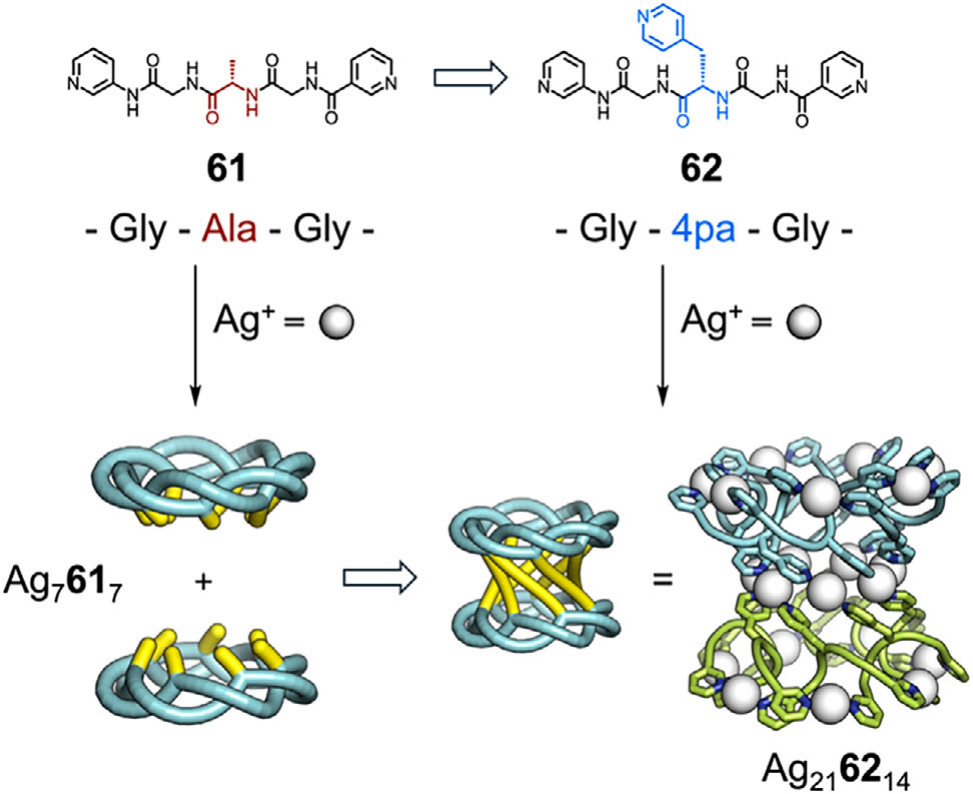
Precise modification of peptide sequence of **61** to form **62** enabling linkage of two Ag_7_ **61**_7_ torus knots into larger structure Ag_21_ **62**_14_.^[^ ^159^ ^]^ Adapted from Ref.[159] Copyright © 2025 Sawada et al.

On addition of Ag(I) these peptides assembled into discrete 12‐crossing [4]catenanes, with differing ring‐crossing topologies depending on sequence. The ditopic pentapeptide Pro–Pro–*x*–Ala–Pro **58** (*x* = an imino‐(1,3‐phenylene) carbonyl spacer) assembled with Ag(I) to form a [6]catenane Ag_24_ **58**_24_ with four interlocking Ag_4_ **58**_4_ macrocycles (Figure 11c).^[^ ^158^ ^]^ The imino‐(1,3‐phenylene)carbonyl spacer enabled formation of an “extended Ω‐loop” conformation, and so favored formation of discrete metallosupramolecular species. The observed [6]catenane structure contained a large cavity, and the Ala in the sequence could be exchanged with a variety of amino acids such as Leu, Gln, and protected Asp and Lys without [6]catenane formation being disrupted. This functional group tolerance was used to explore host–guest chemistry—when the [6]catenane was formed with a peptide in which Ala was replaced by l‐citrulline (**59**), the urea groups presented to the inside of the cavity enabled binding of a 1,3,5‐benzenetriacetic acid guest (Figure 11d). Fujita and Sawada have extensively studied the formation of torus knots from flexible peptide motifs. They hypothesized the arrangement of sidechains in the (*P*)‐form torus knot Ag_7_ **61**_7_ could be used to form higher‐order structures. Changing the sequence of **61** from Gly–Ala–Gly to Gly–4pa–Gly (4pa = 4‐pyridylalanine) (**62**), introduced additional metal coordinating units that could be used to link two torus knots into helical, tubular structure Ag_21_ **62**_14_ (Figure 12).^[^ ^159^ ^]^ Comparisons can be made to protein engineering in which precise “point mutations” in sequence can be used to affect properties without perturbing the base structure.

**Figure 13:**
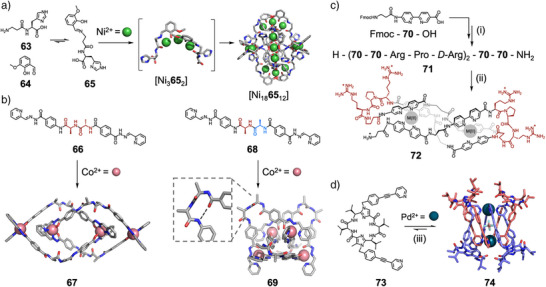
a) Self‐assembly of peptide **65** into enantiopure [Ni_3_ **65**_2_]_6_ assembly.^[^ ^160^ ^]^ Reagents and conditions: Ni(ClO_4_)_2_, Et_3_N, 9:1 MeOH:H_2_O. b) Peptides **66** and **68** and their self‐assembly into contrasting catenane structures **67** and **69**, with key fold‐promoting hydrogen bond in **69** highlighted.^[^ ^162^ ^]^ Reagents and conditions: Co(ClO_4_)_2_, DMSO. c) Synthesis and folding of helicate **72**. Reagents and conditions: i) SPPS, ii) (NH_4_)_2_Fe(SO_4_)_2_ or Co(ClO_4_)_2_, pH 6.5 phosphate buffer.^[^ ^165^ ^]^ d) Self‐assembly of Pd_2_L_4_ lemniscate **74**, with ligands in the crystal structure colored for clarity. Reagents and conditions: iii) Pd(CH_3_CN)_4_(BF_4_)_2_, *d*_3_‐MeCN.^[^ ^169^ ^]^

Similar approaches can be used for multiple metals and peptide structural motifs. Kong and coworkers have recently reported that [Ni_3_] clusters could help fold flexible peptide ligand **65** into a [Ni_3_ **65**_2_] V‐shaped scaffold, which formed a larger enantiopure [Ni_3_ **65**_2_]_6_ assembly (Figure 13a). By changing solvent, smaller [Ni_3_ **65**_2_]_3_ and [Ni_3_ **65**_2_]_2_ assemblies were prepared.^[^ ^160^ ^]^ This approach has been further used to synthesize larger Ni_45_L_30_ assemblies.^[^ ^161^ ^]^ Davis, Shi, Dong, Cui, and coworkers reported a mixed chirality strategy for synthesizing highly folded metal‐peptidic nanostructures.^[^ ^162^ ^]^ They designed two peptide‐based linkers, one homochiral **66** and one heterochiral **68**, both of which contained chelating sites at the *C‐* and *N*‐termini, to facilitate the formation of metal‐peptidic complexes (Figure 13b). Both ligands assembled to form metallocatenane architectures with Co(II); however, the heterochiral system generated a unique 3D interlocked structure (**69**) in which the ligand adopted a folded, *β*‐hairpin‐like conformation due to intramolecular hydrogen bonding. Notably, this structure exhibited substantially different properties to its unfolded counterpart, with enhanced interlocking stability, guest binding affinity, and antimicrobial activity. Additionally, the systems showed significant differences in higher‐order organization, with the heterochiral system displaying remarkable mechanical rigidity in the solid state, demonstrating the important influence of chirality on peptide self‐assembly.

Peptide secondary structure has also been exploited in the design of metallosupramolecular helicates. Vasquez and coworkers have developed synthetic amino acids that are analogues of the common metal chelator 2,2′‐bipyridine, suitable for incorporation in peptides via SPPS.^[^ ^163^, ^164^ ^]^ Six such residues (**70**) were incorporated into 12mer peptide **71** (Figure 13c).^[^ ^165^ ^]^ This peptide contained three sets of two consecutive **70** residues, separated by l‐Arg‐l‐Pro‐d‐Arg sequences, which form *β*‐turn motifs. These *β*‐turn units act as loops which pre‐organize the peptide to form a discrete dinuclear helicate **72** upon addition of Fe(II) or Co(II), as opposed to larger or polymeric complexes. As well as pre‐organizing the ligand's conformation, the peptide sequence directed the chirality of the helicate. Peptide **71**, containing l‐proline, formed the (*Λ*,*Λ*) helicate, while an analogous peptide containing d‐proline formed the (*Δ*,*Δ*) helicate. This highlights the advantages of using inherently chiral and interchangeable amino acid building blocks, as changing peptide sequence enabled ready control of the chiral configuration around metal centers, which can be challenging to achieve.^[^ ^166^ ^]^ Furthermore, the modularity was crucial to optimize the helicate for in vitro and in cellulo investigation, as selective binders of DNA three‐way junctions. The use of l‐Arg‐l‐Pro‐d‐Arg or d‐Arg‐d‐Pro‐l‐Arg sequences to define the *β*‐turns improved solubility, stability, and minimized aggregation compared to previously synthesized helicates with Gly–Pro–Gly defined *β*‐turns. The modularity of peptide synthesis, therefore, allowed individual components of the system to be fine‐tuned to change specific properties.

Wennemers and coworkers have extensively explored the use of oligoprolines and triple helices in both discrete and extended systems, showcasing the advantages of building with biological building blocks by creating platforms for length‐controlled oligomerization and extended triaxial supramolecular weaves.^[^ ^167^, ^168^, ^169^, ^170^ ^]^

Only recently has the use of defined peptide structures, in place of rigid and flat aromatic panels, to form metal‐organic cages been investigated. Metal‐organic cages are of particular interest, as they contain cavities of significant size and variability, capable of binding a wide array of guests.^[^ ^171^ ^]^ Therefore, using modular peptide subcomponents in cage assembly could provide a method to readily tune cavities, and so control host–guest chemistry.^[^ ^172^ ^]^ However, examples of such structures are rare. Clever and coworkers have demonstrated that cyclic pseudopeptide **73**, with imidazole components *N*‐functionalized by pyridyl appended “arms”, self‐assembles on addition of Pd(II) to form an unexpected Pd_2_L_4_ mechanically interlocked lemniscate structure **74** (Figure 13d).^[^ ^173^ ^]^ This unusual structure was templated by an anion within the cage cavity and stabilized by the “basket” conformation of the cyclic pseudopeptide. This allowed *π*‐stacking of the pyridyl arms, and created a cavity for the coordinating pyridines to interact with the peptide backbone. Kubik and coworkers have used V‐shaped cyclic tetrapeptides to assemble Pd_2_L_2_ macrocycles and Pd_3_L_6_ cages.^[^ ^174^ ^]^ In these examples, however, the peptide backbones provide limited scope for derivatization, and have minimal secondary structure.

**Figure 14:**
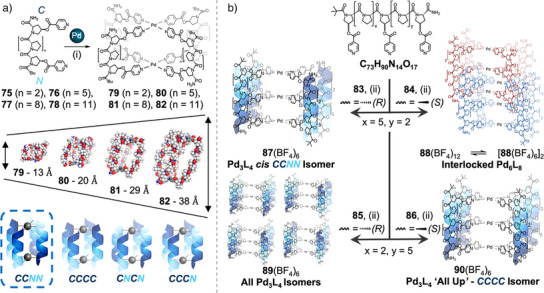
a) Synthesis of Pd_2_L_4_ metal‐peptidic cages. Reagents and conditions: i) Pd(CH_3_CN)_4_(BF_4_)_2_ or Pd(NO_3_)_2_, D_2_O, RT. Molecular models showing family of different sized cages and isomer selectivity in cage assembly.^[^ ^175^ ^]^ Adapted from Ref.[175] Copyright © 2024, McTernan et al. b) Synthesis of Pd_3_L_4_ metal‐peptidic cages showing differing outcomes from four isomeric ligands.^[^ ^176^ ^]^ Reagents and conditions: ii) Pd(CH_3_CN)_4_(BF_4_)_2_, D_2_O, RT.

To overcome this, we have used the rigid and predictable geometry of oligoproline helices as a platform to synthesize adaptable Pd_2_L_4_ cages (Figure 14a).^[^ ^175^ ^]^ Oligoprolines form a robust and rigid PPII helix in aqueous solution, where every third residue is aligned with a separation of 9 Å. This defined geometry can be used for cage self‐assembly; by installing commercially available hydroxyproline (Hyp) at positions *n* and 3*n + *1 in a Pro chain, the PPII fold aligns Hyp residues on the same helical face. Following one‐step esterification of Hyp, to generate parallel coordination vectors with isonicotinic units, rapid self‐assembly of metal‐peptidic Pd_2_L_4_ cage complexes **79**–**82** was observed. By changing the length of the oligoproline sequences, adding or removing triproline repeat units each time (readily achievable by SPPS), the cage length could be varied from ∼1 to 4 nm. Furthermore, ^1^H NMR studies confirmed the selective formation of a single head‐to‐tail isomer, the *cis CCNN*, out of four possible isomers, as the dominant product in solution. Controlling head‐to‐tail isomerism in Pd_2_L_4_ cages has previously proven challenging, with specific modifications of metal binding sites and other strategies required; here, however, the use of complex chiral building blocks provided isomer control as an emergent property of the system. Furthermore, using biologically compatible building blocks allowed us to bind to a range of biologically relevant cargo—including antibiotics, antivirals, and chemotherapy agents—providing a potential route to targeted drug delivery. We then demonstrated that installation of an additional metal‐binding motif in the center of a 10 residue oligoproline ligand enabled formation of Pd_3_L_4_, dual‐cavity, anisotropic “peanut” cages.^[^ ^176^ ^]^ SPPS enabled ready variation of the sequence isomer and/or the stereochemistry of the 4*R*/*S* hydroxyproline to form four novel ligands. On self‐assembly, these generated four distinct outcomes, assembly of Pd_3_L_4_ *cis CCNN* cage isomer **88**, an interpenetrated Pd_6_L_8_ cage **89**, a mixture of all possible isomers of Pd_3_L_4_ cages **90**, or Pd_3_L_4_ “All Up” *CCCC* cage isomer **91** (Figure 14b). These examples show that simple and subtle changes to a peptidic ligand can give rise to complex behavior in metal‐peptidic cage self‐assembly. Palma and coworkers have since highlighted the robustness of our proline platform, by demonstrating analogous cages with our ester Hyp‐pyridine linkers substituted for ether linkers.^[^ ^177^ ^]^

## 5 Conclusions and Outlook

Amino acids and peptides are a vital language in biology and a readily accessible resource for supramolecular chemists. Herein, we began by discussing examples of discrete supramolecular systems with peptides attached and/or incorporated to enhance their biological functions. We then discussed how the fundamental design principles used in supramolecular chemistry apply across length scales, with examples from protein engineering demonstrating the construction of discrete supramolecular systems from whole proteins. Finally, we returned to peptide‐based supramolecular systems, which embrace the folded 3D structure of peptides and so their intrinsic advantages. We hope to have shown how the intrinsic properties of peptides and their folded 3D structures provide a powerful argument for their wider use in supramolecular chemistry.

Synthetic supramolecular systems have, to date, been unable to match the specificity vital to the concerted processes of biology or to operate in crowded milieus such as the cytoplasm. We argue that this is due to the ubiquity of simple organic and conjugated building blocks, with limited information content, in supramolecular chemistry. The incorporation of biological moieties with greater information content and chiral structure will provide a route to greater function and utility. With a large and ever‐growing array of structural biology data providing insight into how protein structures and interactions are controlled, biological systems can provide a wealth of inspiration for synthetic efforts.

We believe that embracing the complex, chiral, and 3D language of biology will unlock a paradigm shift in the magnitude of biological challenges that supramolecular chemistry can address. Peptides provide an ideal middle ground between biological complexity and synthetic accessibility, and their effective use will be key to advancing the field. Peptide secondary structures can be considered scaffolds in which specific functional groups are held in defined orientations from the main helical chain. This enables access to a far richer spatial and functional space but dictates that the field move away from incorporating fragments of peptides in large synthetic systems to understanding how folded peptides can be channeled as scaffolds to form discrete supramolecular structures. Furthermore, complex noncovalent interactions can lead to unexpected structure and function, and so require the design of supramolecular systems that accommodate and exploit the intrinsic properties of the peptide rather than fighting against them. The inherent chirality, tunability, biocompatibility, and robustness of peptides provide an exciting opportunity for supramolecular chemistry, which we believe will be of increasing importance in future research.

## Author Contributions

Authorship is alphabetical between Ben E. Barber, Ellen M. G. Jamieson and Leah E. M. White. *Conceptualization*: Ben E. Barber, Ellen M. G. Jamieson, Leah E. M. White, and Charlie T. McTernan. *Resources*: Charlie T. McTernan. *Writing—original draft*: Ben E. Barber, Ellen M. G. Jamieson, and Leah E. M. White. *Writing—review and editing*: Ben E. Barber, Ellen M. G. Jamieson, Leah E. M. White, and Charlie T. McTernan. *Visualization*: Ben E. Barber, Ellen M. G. Jamieson, Leah E. M. White, and Charlie T. McTernan. *Supervision*: Charlie T. McTernan. *Project administration*: Charlie T. McTernan. *Funding acquisition*: Charlie T. McTernan.

## Conflict of Interests

The authors declare no conflict of interest.

## Data Availability

No primary research results, software or code have been included and no new data were generated or analysed as part of this review.
